# Phrenic nerve afferents elicited cord dorsum potential in the cat cervical spinal cord

**DOI:** 10.1186/1472-6793-5-7

**Published:** 2005-05-06

**Authors:** Yang-Ling Chou, Paul W Davenport

**Affiliations:** 1 Department of Physiological Sciences, Box 100144, HSC, University of Florida, Gainesville FL 32610, USA

## Abstract

**Background:**

The diaphragm has sensory innervation from mechanoreceptors with myelinated axons entering the spinal cord via the phrenic nerve that project to the thalamus and somatosensory cortex. It was hypothesized that phrenic nerve afferent (PnA) projection to the central nervous system is via the spinal dorsal column pathway.

**Results:**

A single N1 peak of the CDP was found in the C4 and C7 spinal segments. Three peaks (N1, N2, and N3) were found in the C5 and C6 segments. No CDP was recorded at C8 dorsal spinal cord surface in cats.

**Conclusion:**

These results demonstrate PnA activation of neurons in the cervical spinal cord. Three populations of myelinated PnA (Group I, Group II, and Group III) enter the cat's cervical spinal segments that supply the phrenic nerve

## Background

The diaphragm is innervated by the phrenic nerve. The well-studied motor innervation of the diaphragm by the phrenic nerve arises from motor neurons in the ventral horn of the cervical spinal cord (Jammes et al., 1995). The phrenic nerve motor innervation originates from C4 to C8 spinal segments in the cat. The diaphragm also has afferent innervation carried to the central nervous system by the phrenic nerve. There are both myelinated and non-myelinated afferents in the diaphragm. The myelinated afferents have conduction velocities consistent with Group Ia, Ib and II afferents. The diaphragm has relatively few Group Ia muscle spindles but a relatively large percentage of Group Ib golgi tendon organs (Duron et al., 1978; Corda et al., 1965a; Goshgarian et al., 1986). Group II mechanoreceptors have also been reported (Corda et al., 1965a). Thus, the diaphragm has innervation with afferents that provide muscle mechanical feedback to the CNS via the phrenic nerve. However, physiological evidence of phrenic afferent activation of the spinal cord dorsal horn is lacking.

The role of phrenic afferents in the regulation of diaphragm function has been studied with early observations suggesting that phrenic afferents in the diaphragm are not involved in controlling the respiratory muscle activity (Sant'Ambrogio et al., 1962; Corda et al., 1965b; Kohrman et al., 1947; Landau et al., 1962). However, electrical and mechanical stimulation of phrenic afferents are reported to activate thalamic neurons (Zhang et al., 2003) and elicit neural activity in the cat somatosensory cortex (Davenport et al., 1985). In addition, Zechman et al (1985) reported a correlation of transdiaphragmatic pressure (Pdi) and the perception of inspiratory loads. Knafelc and Davenport reported that increased Pdi correlated with the amplitude of the respiratory related evoked potential recorded from the human somatosensory cortex. Thus, there appears to be a projection of diaphragmatic afferents to the CNS and mechanical changes in the diaphragm correlate with somatosensory activation of the cerebral cortex. However, PnA activation of spinal sensory pathway(s) remains unknown.

Anatomical studies have found that phrenic nerve afferents (of unknown type) terminate in the dorsal horn lamina I-IV of C4 and C5 spinal cord in rat (Goshgarian et al., 1986; Malakhova et al., 2001). In a brief report, Larnicol et al (1984) reported immunoflourescent evidence of dorsal root entry of phrenic nerve afferents in the dorsal horn of the cervical spinal cord. Gill et al (1963) also demonstrated phrenic motorneuron activities were elicited by segmental phrenic nerve afferents. Corda et al (1965) demonstrated that the cervical spinal dorsal rootlets contain diaphragmatic mechanoreceptors, including muscle spindles and Golgi tendon organs. Electrophysiological studies have confirmed that Group I and Group II phrenic nerve afferents project to lateral reticular nucleus (Macron et al., 1985), external cuneate nucleus (Marlot et al., 1985), and both ventral and dorsal respiratory-related areas of brainstem (Macron et al., 1986; Speck et al., 1987). Moreover, in anesthetized cats, short-latency responses have been recorded in the thalamus (Zhang et al., 2003) and somatosensory cortex (Davenport et al., 1985) after electrical stimulation of the phrenic nerve afferents and mechanical probing of the diaphragm. If diaphragmatic proprioceptors project to higher somatosensory brain centers via pathways similar to Group Ia and Group Ib receptors (Landford and Schmidt, 1983), then phrenic afferents should enter the spinal cord ipsilaterally through the dorsal roots of the cervical spinal segments, terminate on dorsal horn neurons, dorsal horn neurons should then project centrally via the dorsal columns to the brainstem and then project to the somatosensory cortex and other supraspinal structures. If this is the phrenic afferent pathway to the somatosensory cortex, then electrical stimulation of the phrenic nerve will stimulate phrenic afferents and activate dorsal horn neurons. However, the activation of the cervical dorsal horn by stimulation of phrenic afferents has not been reported.

One method to investigate dorsal horn neuronal activation by afferent stimulation is recording the cord dorsum potential (CDP) (Yates et al., 1985). Simultaneous stimulation of peripheral afferents activates groups of dorsal horn neurons. The activation of a group of neurons produces a dipole referenced to the surface of the spinal cord. This dipole is the result of a change in polarity of the activated neurons creating a current flow with the cord surface. Thus, neurons in the dorsal horn of the spinal cord generate a field potential when an afferent volley arrives. These negative voltage evoked potentials generated from the dorsal horn neurons of the spinal cord by stimulation of peripheral nerves have been extensively studied for limb afferents (Yates et al., 1982; Manjarrez et al., 2002). The CDP was first discovered and described by Grasser and Graham (1933) as they recorded complex evoked potentials from the surface of the spinal cord. This potential appeared to be largest in the dorsal horn grey matter and is generated by a synchronous activation of a population of dorsal horn neurons that respond to stimulation of low-threshold cutaneous afferents.

Stimulation of nerve afferents at an intensity that activates only Group I muscle afferents has been shown to evoke a dorsal cord field potential consisting of a triphasic spike, a short duration negative wave, and a positive wave. Activation of Group II muscle afferent fibers resulted in a second short duration negative component of the CDP (Bernhard, 1953; Coombs et al., 1956). When a nerve is stimulated sufficiently distal to the spinal cord, the depolarization of the dorsal horn neurons occurs sequentially as a function of the arrival of afferents with different conduction velocities. Thus, it has been shown that the triphasic spike of the CDP occurs because of a separation of activation due to the arrival of Group Ia, Group Ib and Group II afferents. The different peak latencies allow for the determination of different populations of activated afferents.

We reasoned that, if the phrenic nerve contains Group Ia, Group Ib and Group II afferents, then stimulating the phrenic nerve as far distal from the spinal cord as possible (near the diaphragm) would elicit multiple CDP peaks. In addition, we hypothesized that if phrenic nerve afferents enter a cervical segment of the spinal cord, then stimulating PnA will elicit a CDP in that segment. However, the CDP for phrenic nerve afferents has never been reported. Therefore, recording the phrenic afferent CDP was hypothesized to provide evidence of segmental dorsal horn activation by phrenic afferents. This study recorded the CDP from C4 to C7 elicited by stimulating PnA to determine the cervical spinal segmental distribution of PnA elicited CPD, to characterize CDP latencies and infer the populations of PnA eliciting the CDP in cats.

## Results

**Figure 1:**
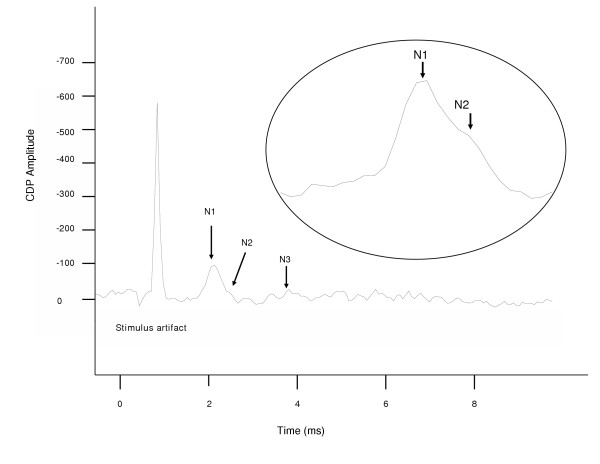
The cord dorsum potential in response to phrenic nerve afferent stimulation in the C5 segment. The trace was the average of 32 stimulus epochs for the middle C5 segment from one animal. The enlarged area expands the large initial peak of the CDP to better illustrate the N1 and N2 peaks. The first negative wave indicates the electrical stimulus used as the zero time point for peak latency analysis. The stimulus artifact is followed by three negative peaks N1, N2, and N3. The amplitudes of the negative peaks were measured from the voltage difference between the peak voltage and the averaged voltage of the 5 ms period before the stimulus onset.

**Figure 2:**
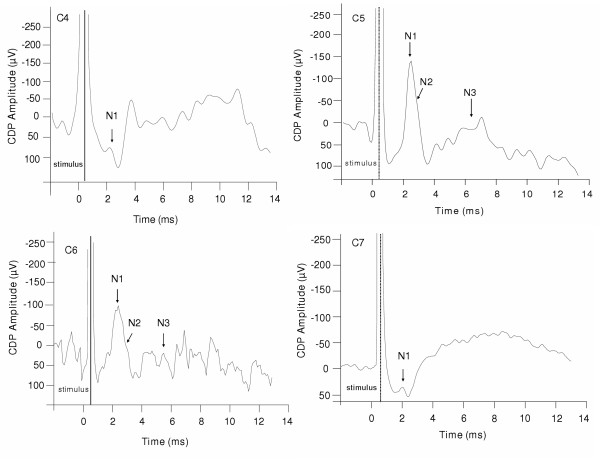
Representative illustration of phrenic nerve afferents stimulation related cord dorsum potential recorded at C4, C5, C6, and C7 spinal segments in one animal. The traces were the average of 32 stimulus epochs for each spinal segment. The N1 peak was recorded in all cervical spinal segments (C4 to C7); whereas N2 and N3 CDP peaks were identified only in C5 and C6 spinal segments. The amplitudes of the negative peaks were measured from the voltage difference between the peak voltage and the averaged voltage of the 5 ms period before the stimulus onset.

**Figure 3:**
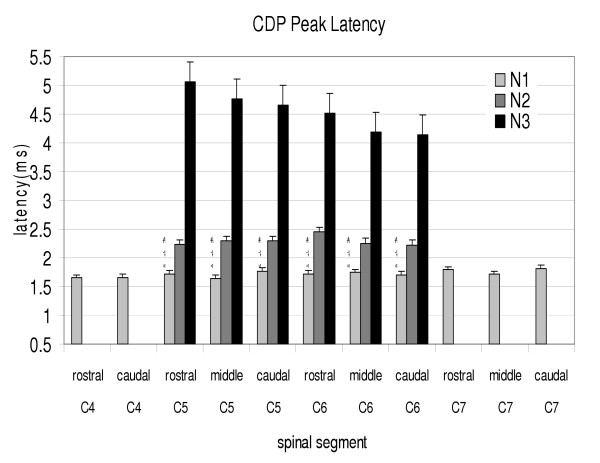
Histogram of the CDP peak latencies in different regions of the cervical spinal segments (C4 to C7) after phrenic nerve afferents stimulation in cats. The intensity of the stimulation was 500 μA. N1 is the first peak latency. N2 and N3 are the second and third peak latencies found in C5 and C6 only. * indicates a significant differences between N1 and N2 peaks. † indicates a significant difference between N2 and N3 peaks in the C5 and C6 segmental locations. # indicates a significant difference between N1 and N3 peaks in the C5 and C6 segmental locations.

**Figure 4:**
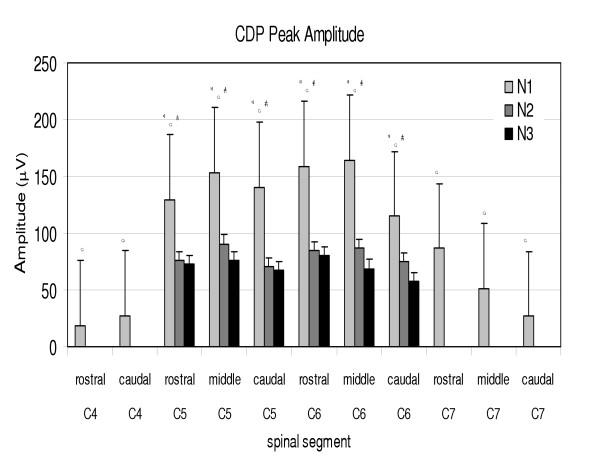
Histogram of the CDP peak amplitudes in the cervical spinal segments (C4 to C7) after phrenic nerve afferent stimulation in cats. N1 is the first peak amplitude observed in the C4 to C7 spinal segments; N2 and N3 are the second and third peak amplitudes only observed in the C5 and C6 spinal segments. * indicates a significant differences between N1 and N2 peaks in the C5 and C6 segmental locations. # indicates a significant difference between N1 and N3 peaks in the C5 and C6 segmental locations. ∞ indicates significant differences between the spinal segments for N1 peak.

In all cats, electrical stimulation of the phrenic nerve elicited CDP's recorded at the dorsal surface of C4 to C7 cervical spinal segments, and at rostral, middle and caudal locations within each spinal segment. No CDP was observed in the C8 spinal segment. A primary CDP elicited by stimulation of PnA was observed and recorded in dorsal surface of C4 to C7 cervical spinal segments (Fig. 1). The N1 CDP was recorded in the C4 to C7 spinal segments; whereas three CDP peaks (N1, N2 and N3) were identified only in the C5 and C6 spinal segments (Fig. 2). The distributions of the onset latencies and peak amplitudes of individual cervical spinal cord segments are summarized in (Fig. 3) and (Fig. 4), respectively. The averaged N1 peak latency was 1.7 ± 0.1 ms for all cervical spinal segments. The averaged N2 peak latency was 2.3 ± 0.1 ms and the averaged N3 peak latency was 4.6 ± 0.3 ms in the C5 and C6 segments. The averaged conduction velocity was 94.1 ± 8.6 m/sec for the N1 peak, 70.6 ± 7.5 m/sec for the N2 peak and 35.0 ± 3.8 m/sec for the N3 peak. There was a significant difference between the latencies for N1, N2 and N3 peaks (P < 0.05). The average N1 peak amplitude was 22.8 ± 6.0 μV in the C4 segment and significantly less than the N1 peak amplitude for C5, C6 and C7 spinal segments (p < 0.05). The average N1 peak amplitude was 141.3 ± 12.1 μV in the C5 segment and 146.0 ± 27.2 μV in the C6 segment and not significantly different. The average N1 amplitude was 54.6 ± 30.1 μV in the C7 segment and significantly less than C5 and C6. N2 and N3 peaks were only observed in the C5 and C6 spinal segments and the averaged N2 peak amplitude was 79.2 ± 10.3 μV in the C5 segment and 82.1 ± 6.5 μV in the C6 segment. The averaged N3 peak amplitude was 72.0 ± 4.4 μV in the C5 segment and 69.0 ± 11.3 μV in the C6 segment. The amplitudes of the CDP peaks were significantly different between the N1, N2 and N3 peaks (P < 0.05).

## Discussion

Neural activity was elicited by stimulation of PnA in C4 to C7 dorsal cervical spinal cord segments in the present study. The electrical stimulation of PnA served the important purpose of demonstrating the existence and locations of PnA- elicited CDP in C4 to C7 segments of the cat cervical spinal cord. This stimulation of PnA was initially observed by one or three negative peaks depending on different segments recorded. The presence of the three negative peaks in C5 and C6 spinal segments appears to be due primarily to the spinal input from different groups of PnA. The presence of only the N1 peak in C4 and C7 demonstrates a different number of afferent populations between the cervical spinal segments that contribute to the cat phrenic nerve.

### Central projections of PnA

CDP recordings are evidence of the activation of neurons in the dorsal horn of the cervical spinal cord by PnA. Neural activity in the dorsal surface of the cervical spinal cord elicited by PnA found in this study is consistent with the previous studies of limb muscle afferents (Yates et al., 1985). These results are consistent with PnA entering the cervical spinal cord, projecting to the dorsal horn, which then relays PnA to the brainstem, and projects phrenic afferent information to the somatosensory cortex via a thalamocortical pathway (Zifko et al., 1995; Davenport et al., 1985; Zhang et al., 2003). The function of this putative pathway could be related to the proprioceptive control of respiratory muscles and respiratory movement control originating in the motor cortex (Frazier et al., 1991). Davenport et al (1985) proposed that the PnA projection to the postcruciate region of the cerebral cortex may play a role in higher brain center control of the respiratory pump. The sensory projection sites of the PnA were localized in area 3a and 3b of the sensorimotor cortex in cats. However, the PnA sensory sites in the cortex are not co-localized with motor sites. This means that the cortical regions receiving the sensory information from PnA are separated from the cortical regions of motor output to the phrenic spinal motor neurons. Recordings of evoked potentials using phrenic nerve stimulation provide a unique method for studying the potential pathways for cortical integration of respiratory afferent information and the projections of PnA to the central nervous system (Straus et al., 1997). Although the PnA projection pathways to the cerebral cortex remain unknown, the present study supports a dorsal column mediated pathway that likely involves a multisynaptic thalamic relayed projection (Zhang et al., 2003). It has been shown in the previous studies (Frazier et al., 1991) that C-fiber afferents have much higher threshold and longer latency for PnA stimulation than group Ia, Ib and group II afferents. Conduction velocities for C-fibers are less than 1 m/s. With the length of the phrenic nerve for these cats, the latency for a C-fiber elicited peak of the CDP would be approximately 30 ms, longer than the 25 ms sampling time used for recording the post-stimulus epochs in this study. In addition, the use of 0.1 ms stimulus pulse width does not elicit c-fiber action potentials. Therefore, it is unlikely that C fiber afferents contributed to the CDP in this experiment.

### PnA elicited CDP

Results of this study provide evidence that a spinal CDP was elicited by phrenic nerve afferents input to the dorsal horn of the cat cervical spinal cord. This result is consistent with the report from Cuddon et al (1999) that the CDP is a measurement of spinal segmental interneurons and dorsal horn cell function. The rationale behind the CDP measurement as an assessment of sensory nerve afferent and dorsal horn neuronal functions is the phases of the CDP reflect the different population of afferent fibers activating dorsal horn neurons (Yates et al., 1982; Yates et al., 1985). The large negative peak (N1) represents the interneuronal depolarization of spinal dorsal horn neurons elicited by large myelinated sensory afferents (Group Ia & Ib). The activation of a N1 peak in C4 to C7 spinal segments suggests a broadly distributed input to the spinal cord from phrenic group I afferents (Corda, et al., 1965). A N1 peak was not recorded in the C8 spinal segment. There is a second negative peak (N2) that represents the depolarization of spinal dorsal horn neurons by slower conducting myelinated sensory afferents (Group II). A third negative peak (N3) most likely represents the depolarization of spinal dorsal horn neurons elicited by small myelinated sensory afferents (Group III). The presence of N2 and N3 in the C5 and C6 spinal segments suggests a preferential input of group II and group III afferents into these specific spinal segments.

Limb group Ia, Ib and II have been shown to project to somatosensory cortex of cat via a dorsal column pathway (Jones et al., 1980). Corda et al (1965) reported group Ia, Ib and II afferents in the phrenic nerve. It is very likely that PnA have a projection similar to limb proprioceptors. This conjecture is supported in the present study by the CDP elicited in the dorsal surface of cervical spinal cord by stimulation of PnA which is consistent with a dorsal column central neural projection pathway. It is therefore concluded that group Ia, group Ib, group II and possibly group III afferents elicit a CDP that is consistent with an ascending phrenic sensory pathway via the dorsal column and the dorsolateral funiculus (spinocervical tract) of the cervical spinal cord.

### Somatosensory PnA pathway

Cortical projection of PnA has been shown in both cortical evoked potentials (Zhang et al., 2003; Davenport et al., 1985) and retrograde fluorescent (Yates et al., 1987) studies. Activation of the somatosensory cortex in cats after electrical stimulation of the contralateral phrenic nerve (Davenport et al., 1986) and intercostals muscles (Davenport et al., 1993) has been reported by this laboratory. One role of the somatosensory projections from phrenic nerve afferents may be to provide the sensory feedback to the cerebral cortex of respiratory pump function. The diaphragm, the intercostal muscles and accessory muscles of respiration provide the inspiratory pumping force for ventilation. Stimulation of PnA has been shown in humans to elicit somatosensory cortical evoked potentials (Zifko et al., 1996). Inspiratory occlusion produces a maximal load on the pumping action of the respiratory muscle and has been reported to elicit somatosensory respiratory related evoked potential (RREP) in humans (Davenport et al., 1986; Davenport et al., 2000) and lambs (Davenport et al., 2001). Knafelc and Davenport reported a correlation between RREP amplitude and the magnitude of the increase in Pdi when graded inspiratory resistive loads were applied in humans. These reports suggest that mechanical loading of the respiratory muscles, including the diaphragm, can elicit somatosensory cortical neural activity. Although the afferents mediating these evoked potentials are unknown, it is likely that respiratory muscle afferents are one population of receptors that mediate these responses. The results presented in the present study are therefore consistent with the hypothesized role of PnA in the somatosensation of inspiratory loads. Thus, neurons in dorsal spinal cord activated by stimulation of PnA may be related to respiratory muscle proprioception, similar to what has been found in other muscle systems.

## Conclusion

In summary, the present study recorded a spinal CDP elicited by the activation of phrenic nerve afferents. The PnA project to dorsal horn neurons in the cervical spinal cord from C4 to C7 indicating these segments can function as a relay for the conduction of proprioceptive information from the diaphragm to the higher brain centers in cats. The first peak, N1, conduction velocity is consistent with large myelinated afferent activation, group Ia and group Ib. These PnA enter all the spinal segments that contribute to the phrenic nerve in cats. The second peak conduction velocity is consistent with myelinated afferents, group Ib and large group II. The third peak conduction velocity is consistent with myelinated afferents, group III. The second and third peaks of the CDP were observed only in the primary spinal origin (C5 and C6 segments) of the cat phrenic nerve. The CDP potentials described in this study reflect the first relay by the dorsal spinal cord of the projection of myelinated PnA to the higher brain centers in cats. Therefore, the results of this study support the hypothesis that PnA activation of neurons in the dorsal cervical spinal cord may be involved in the central projection of respiratory muscle afferent information.

## Methods

### General preparation

Experiments were carried out in adult cats (2.5–3.0 Kg). The University of Florida, Institutional Animal Use and Care Committee reviewed and approved this study. CDP recordings were made from cervical spinal segments C4, C5, C6, C7, and C8 in anesthetized, paralyzed, and artificially ventilated animals. Adult cats (n = 7) of either sex were anesthetized with inhalation of halothane-oxygen. The femoral artery and vein were catheterized. Gas anesthesia was then replaced with α-chloralose by slow i.v. infusion (25 mg/ml). The animals were tracheotomized, vagotomized and placed prone with the head and spine fixed into a stereotaxic apparatus. The body temperature was monitored with a rectal probe and maintained at 38° ± 1°C with the periodic use of a heating pad. Arterial blood pressure, expired CO_2 _and tracheal pressure were continuously monitored on a polygraph. The animals were connected to a mechanical ventilator and paralyzed (gallamine triethiodide). If fluctuation in blood pressure and heart rate were observed, all experimental procedures were suspended and supplemental anesthesia was administered (0.1 mg/kg iv per dose) until a surgical plane of anesthesia was reestablished. The lungs were periodically inflated to prevent atelectasis. Arterial blood gases and pH were measured and maintained within the normal range. The animals received a continuous infusion of lactated Ringers.

### Protocol

The skin and muscles overlying the right lower ribs were incised. The 7^th ^intercostal space was identified and opened by cutting the intercostal muscles. The intercostal space was opened with retractors. The right phrenic nerve caudal to the heart was identified, isolated and dissected free of the surrounding tissue. The phrenic nerve, about 1 cm cranial to its entry into the diaphragm, was placed across bipolar platinum stimulating electrodes. The cathode electrode was about 5–7 mm proximal to the anode electrode. Supramaximal single pulse stimuli (250–500 μA) were delivered at a rate of 0.6 Hz with stimulus duration at 0.1 milliseconds. The pulse simultaneously triggered the signal averager for collecting the 25 msec. post-stimulus spinal activity sample. The entire surgical field was covered with saline soaked gauze.

Spinal laminectomies were performed to expose the dorsal spinal cord from C_2_-T_2_. The dura was reflected. The exposed spinal surface was flooded with warm mineral oil. A silver-silver chloride ball electrode was lowered to the pial surface. This electrode was used to record the CDP. The electrode was connected to a high impedance probe, which was connected to an amplifier. The electrical signal was band pass filtered at 3 Hz – 3.0 kHz and amplified. The amplifier output was led into a signal averager (Model 1401, Cambridge Electronic Design, Ltd) At least 64 post-stimulus epochs were sampled at 10 kHz and recorded to provide a minimum of 32 evoked spinal epochs averaged by the computer system to obtain the CDP (Signal2, Model 1401 Cambridge Electronics Ltd.). The recording electrode was placed on the surface of the ipsilateral dorsal spinal cord medial to the dorsal root entry zone of the spinal segments. Each spinal segment was subdivided into 3 recording regions: rostral, middle and caudal based on counting the dorsal roots. The electrode was systematically moved over the spinal surface at each recording site between C4 and C8. The ground electrode was placed over a bony prominence. Phrenic nerve stimulation was performed at each point and the CDP recorded.

### Data analysis

A minimum of 32 evoked epochs were averaged by a computer system (Signal2 Cambridge Electronics Ltd.) to obtain the CDP. The averaged CDP's were analyzed for peak presence and polarity (Signal-2, Cambridge Electronics Ltd). The peaks of the CDP were negative voltage changes that were initially identified in C5 (Fig. 1) and C6 recordings. The peaks were identified and then labeled based on latency ranges from the known conduction velocities of phrenic nerve afferents (Corda et al., 1965) and CDP peak analysis reported for limb afferents (Yates et al., 1982). The corresponding onset and peak latencies and amplitudes were determined. The 0-peak amplitudes were measured from the voltage difference between the peak voltage and the averaged voltage of the 5 msec period before the stimulus onset. The initial post-stimulus baseline was corrected for any DC offset. The means and standard errors for onset and peak latencies and amplitudes were then calculated. The onset latency of each CDP was measured from the onset of the stimulus to the start of first peak of the CDP. The latency of the first negative peak (N1) of each CDP was measured as the time from the stimulus to the peak. When present, the latency of the second negative peak (N2) and the third negative peak (N3) were measured in the same manner. The length of phrenic nerve was measured from the stimulating electrodes to the dorsal root entry zone to calculate the conduction velocity of the phrenic nerve afferents. Each component of CDP was averaged and statistical analysis was applied. The mean ± standard deviation (SD) was calculated for all CDP peak latencies. One-way repeated measure ANOVA was used to compare between the N1 peak, N2 peak, and N3 peak latencies and amplitudes from each recording site. The criterion for significance was p < 0.05.

## Abbreviations

PnA – Phrenic nerve afferents

CDP – cord dorsum potential

C4, C5, C6, C7, C8 – Cervical spinal segments

CNS – Central nervous system

Pdi – Transdiaphragmatic pressure

CO_2 _– Carbon dioxide

## Authors' contributions

YL performed the data analysis and drafted the manuscript. PD carried out the study and coordinated its design. Both authors read and approved the final manuscript.
